# A Fully Integrated Low-Power Multi-Mode RF Receiver for BDS-3/GPS

**DOI:** 10.3390/s23177631

**Published:** 2023-09-03

**Authors:** Shalin Huang, Jiang Li, Mingdong Li, Fang Tang

**Affiliations:** School of Microelectronics and Communication Engineering, Chongqing University, Chongqing 401331, China; shalin_huang@163.com (S.H.); lijiang150@sina.com (J.L.); drlmd1009@126.com (M.L.)

**Keywords:** RF receiver, BeiDou navigation satellite system-3 (BDS-3), global positioning system (GPS), multi-mode receiver

## Abstract

A fully integrated low-power area-efficient receiver using a low–intermediate frequency topology for BDS-3 and GPS L1 bands is presented in this paper. Accurate localization can be achieved without requiring off-chip low-noise amplifiers. The receiver bandwidths for GPS and BDS-3 are 2 MHz and 4 MHz, respectively. Digitally assisted calibration schemes, such as RC calibration, automatic gain control, and DC offset correction are integrated to resist the effects of the process, voltage, and temperature (PVT) variations. The receiver—fabricated in a standard 55 nm CMOS technology—provides a maximum gain of 113.2 dB, a gain control range of 61 dB, and a minimum noise figure of 1.74 dB under a 1.2 V supply. The receiver, with and without the frequency synthesizer that provides the local oscillator frequency, consumes 8.7 mA and 4.8 mA, with areas of 0.73 mm^2^ and 0.345 mm^2^, respectively.

## 1. Introduction

The completion of the BeiDou navigation satellite system-3 (BDS-3) in July 2020 marked a major milestone for China’s satellite navigation system. BDS-3 is designed to provide global coverage, with 35 satellites in orbit, and it offers a range of positioning, navigation, and timing (PNT) services. The system is intended to offer a more reliable and accurate alternative to GPS and several advanced features, such as anti-jamming and anti-spoofing capabilities [1]. The ongoing application development, such as BeiDou+ and +BeiDou, is expected to drive the BDS industry growth further.

With the broad deployment of the Internet of Things (IoT) systems, there is a growing need for navigation terminals/receivers capable of operating for extended periods with low power consumption and device costs. Currently, there are many receivers designed for the global positioning system (GPS), global navigation satellite system (GLONASS), the Galileo system, and so on [2,3,4,5]. However, they are all incompatible with BDS-3 due to the different bandwidth requirements, operating frequencies, and demodulation algorithms. Until now, few CMOS-integrated receiver designs for the BDS-3 system have been reported on [6,7,8,9].

**Figure 1:**
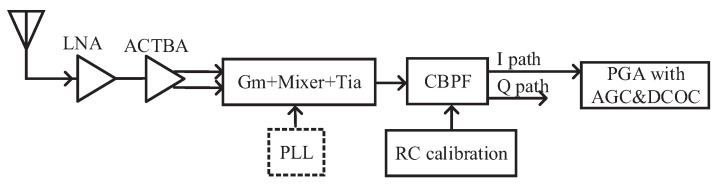
Simplified receiver system diagram.

This paper presents a low-power, area-efficient receiver that is suitable for both BDS-3 and GPS in the L1 band (with a carrier frequency of 1575.42 MHz). Satellite signals from the two systems can be received simultaneously to improve localization speed and accuracy. Specifically, as BDS-3 does not have strict requirements on linearity and image rejection ratio (IMRR) in [10]; this work focuses more on the receiver performance regarding power optimization and area minimization. A simplified RF receiver system of this design is shown in Figure 1, which consumes only 4.8 mA with a small area of 0.345 mm^2^ fabricated in a standard 55 nm CMOS technology. To achieve this performance, the major considerations are listed below.

1. In the on-chip low-noise amplifier (LNA), the source inductor and gate inductor with high Q-value are realized using copper bonding wire. This improves in-band noise performance and saves space. LNA adopts a cascode amplifier structure with the LC resonant network as the load and features reused current, improving the gain and reducing power.
2. Adopting the active balun amplifier (ACTBA) instead of the traditional passive balun to convert the single-ended signal into the differential signal not only improves the RF front-end gain but also reduces the gain-realization difficulty of the later-stage circuit, which is beneficial for achieving high gain and conserving system space. ACTBA employs current reuse to reduce power consumption.
3. A GMT circuit is modified, which can save the intermediate frequency (IF) direct current (DC) blocking capacitors to reduce the insertion loss and area.
4. Image suppression is realized by bandpass filter (BPF), and the later circuits only need to amplify and convert the in-phase channel signals, saving nearly half of the IF layout area.

The rest of this paper is organized as follows. Section 2 analyzes the GPS and BDS-3 signal properties to check the feasibility of receiving both signals simultaneously, as well as the receiver requirements for the BDS-3 system. Section 3 describes the target specification of each sub-circuit of the receiver system, including cascaded gain, dynamic range, noise figure, linearity, and filter order. Section 4 elaborates on the architecture of the receiver and the circuit implementation. Section 5 shows the measurement results, followed by a conclusion in Section 6.

## 2. GPS and BDS-3 Signal Properties

### 2.1. Features of GPS and BDS3

The target receiver should receive both the GPS and BDS-3 satellite signals to improve localization accuracy. The signal properties of the two systems must be analyzed to check the feasibility of receiving both signals simultaneously. According to [11,12], the two systems have the same carrier frequency of 1575.42 MHz and an antenna polarization of right-hand circular polarization (RHCP). Therefore, their RF antenna, local oscillator (LO) frequency, and receiver structure are compatible with each other without additional receiving links. However, they differ in the received power levels and signal bandwidth. In [11,12], the received signal levels (receiver on the earth ground) of GPS and BDS-3 are −127 dBm and −129 dBm, respectively. In terms of bandwidth, BDS-3 requires a width of 4.092 MHz, which is twice that of the GPS. The difference in bandwidth does not affect the RF front-end circuits, but it is necessary to design an analog filter in the receiver with a tunable bandwidth. The in-band thermal noise power can be calculated by $N=N_{T0}\times BW$, where $N_{T0}=kT_{0}$, k is the Boltzmann constant, $T_{0}$ is the room temperature in Kelvin, the value of $N_{T0}$ is usually −174 dBm/Hz [8], and BW is the bandwidth. According to the bandwidths of GPS and BDS-3, the in-band thermal noise power levels are −111 and −108 dBm, respectively. Therefore, the minimum power level arriving at the antenna port for the GPS L1 band is 16 dB lower than the thermal noise. While the BDS-3 signal is about 21 dB lower than the thermal noise. The RF power levels received on the ground are below the thermal noise floor due to the far distance between the satellite and the ground [5]. Therefore, for a receiver to work for both GPS and BDS-3, the compatibility of the bandwidth and cascade gain requirements must be met.

### 2.2. Overall Requirements of the BeiDou Navigation Satellite System-3

The overall requirements for the proposed receiver have been put forward by the China Satellite Navigation Office in their general specifications [10]. Analyzing the overall requirements, the main difficulties in this design are as follows:

1. The RF signal attenuates significantly at a high frequency of 1575.42 ± 2.046 MHz due to the parasitic effect caused by routing and packaging. The transconductance can be increased by enlarging W/L or the current to boost the gain. However, large-sized transistors make the parasitic capacitance influence more serious. A large current results in heightened power consumption. Therefore, it is difficult to realize an RF receiver with high working frequency and low power.
2. In the receiver design, RF circuits with excessive gain lead to greater power consumption and more complex RF layouts. If the gain of the RF circuits is reduced, the gain of the intermediate frequency (IF) circuits must be increased to meet the gain requirements of the system. However, the IF circuits with excessive gain will bring instability to the system. Since the gain of the RF circuits decreases, the noise contribution from the IF circuits increases. Therefore, RF and IF circuits must reasonably allocate the cascade gain to ensure system stability.
3. External LNA [4] is generally used to provide better noise performance in practical applications. However, this external LNA is not expected in our design in the case of weak interference, which puts forward higher requirements for noise performance. The noise figure (NF) will be worsened due to crosstalk and noise coupling on the substrate of the system-on-chip. Moreover, the low-power requirement also limits the further improvement of NF. Therefore, it is difficult to realize the receiver-on-chip with a low NF.
4. An image rejection ratio (IMRR) of 25 dB is required. Generally, increasing the IF or the CBPF order can improve the IMRR, but the current and area increase. So it is not conducive to a low-power design.

Therefore, designing a high-performance BDS-3/GPS receiver that prioritizes low power consumption and occupies a small area is challenging.

## 3. Systematical Design of the Receiver

To match the overall requirements, the key indicators should be properly allocated to each sub-circuit. By making compromises in NF, linearity, filter order, etc., power and area (costs) can be reduced [13]. Next, the allocation of some indicators of the proposed receiver is discussed, and the allocation results are summarized in Table 1, where GMT is the mixing stage, including the Gm, passive mixer, and transimpedance (Tia) amplifier.

### 3.1. Gain and Gain Control Range

As mentioned above, the in-band thermal noise power levels for GPS and BDS-3 are −111 and −108 dBm, respectively. The minimum power level arriving at the antenna port is lower than the in-band thermal noise. Therefore, thermal noise dominates the input power level of the receiver [4,5]. It means that noise determines the cascade gain and analog–digital conversion (ADC) quantization threshold [5]. Assuming that the optimal input signal power of ADC is 0 dBm, the needed cascade gain of the proposed receiver is at least 111 dB. The practice has proved that an RF circuit with a gain of 40∼55 dB is reasonable [5,7,8,14]. The gain of the RF circuit is tentatively set at 51 dB, where the signals can be normally received. A gain control range (Gr) larger than 45 dB needs six-bit control words to be implemented in IF circuits because the corresponding binary of 45 is 101 101. To avoid wasting any resources, the Gr is tentatively set at 63 dB, whose corresponding binary is 111 111. This means that the gain range of IF circuits is between 0 and 63 dB. In all, the maximum cascade gain is tentatively set at 114 dB, considering a safety margin of 3 dB.

### 3.2. Noise Figure

The design strives to not use external LNA in some weak interference scenarios, reducing costs, so the target NF is 2.5 dB, which is lower than the required 5 dB proposed by [10]. The corresponding cascade noise factor is about 1.7783. According to the cascade noise factor equation [15,16] and the system architecture, it is mandatory to increase the gain of the first-stage LNA while keeping its NF as low as possible. So the noise factor of the first-stage LNA is 1.7. The corresponding NF is 2.3 dB. The more backward the sub-circuit, the greater the cascade gain. Hence, the later-stage sub-circuits with larger NFs can reduce the design difficulty. As a result, the noise factors of ACTBA, GMT, BPF, and PGA are 6.31, 22.39, 3162.28, and 3162.28, respectively, and the corresponding NFs are 8 dB, 13.5 dB, 35 dB, and 35 dB, which are summarized in Table 1.

### 3.3. Linearity

Input 1 dB compression point ($IP_{1\mathrm{dB}}$) and input third-order intercept point (IIP3) are both indicators that represent linearity performance. $IP_{1\mathrm{dB}}$ and IIP3 should be greater than −75 dBm and −65 dBm at the minimum cascaded gain according to the general specification [10]. Usually, IIP3 is about 10 dB higher than $IP_{1\mathrm{dB}}$ [17]. Therefore, ${IP}_{1\mathrm{dB}}$ is allotted first in this section. It is supposed that when $IP_{1\mathrm{dB}}$ is −75 dBm, the in-band signal can be linearly amplified by each sub-circuit. The in-band power (IBP) at the output of each sub-circuit is obtained in Table 1. Next, interference is considered. For the BDS-3 and GPS L1 bands, the nearest interference is at 1710 MHz from the LTE channel. According to the technical specification [18] issued by 3GPP, the maximum transmission power is 23 dBm. After a communication distance of 3.5 m with an ideal attenuation of 48 dB, the interference power is −25 dBm, which is so large that it is needed to add an external SAW filter to suppress the out-of-band interference. In order to prevent the SAW filter from worsening the NF, an external monolithic LNA will be added in front of the SAW. So, coupled with a SAW filter (SAFFB1G57KE0F0A of Murata) and an external LNA (SW7121D of Silicon Wave), the interference power arriving at the RF input is less than −56 dBm. When only the RF gain of 51 dB is considered, the out-of-band interference power (OBIP) at GMT’s output is −5 dBm. But after GMT, the interference frequency is 134.58 MHz from the center frequency. So, taking into account the out-of-band attenuation provided by GMT, the OBIP at GMT’s output is less than −25 dBm. An out-of-band rejection of 75 dB at 1710 MHz is easily achieved by CBPF. So the OBIP at the programmable gain amplifier’s (PGA’s) output is less than −100 dBm, which does not affect the linear capability at all. To summarize, when considering in-band signals, $IP_{1\mathrm{dB}}$ should be greater than −75 dBm; when considering out-of-band interference, $IP_{1\mathrm{dB}}$ should be greater than −56 dBm. So, all in all, $IP_{1\mathrm{dB}}$ should be greater than −56 dBm. In other words, as long as the interference power at the RF input is less than −56 dBm, there is no need for the off-chip SAW and LNA.

Combined with previous product experience, the $IP_{1\mathrm{dB}}$ breakdown of each sub-circuit is shown in Table 1, and the cascade $IP_{1\mathrm{dB}}$ is about −51 dBm, considering 5 dB margins, which caters to the requirement in [10]. The IIP3 values for each sub-circuit are also summarized in Table 1. Using the cascade IIP3 calculation equation from [19], the cascade IIP3 is −43.39 dBm.

### 3.4. Filter Order

The frequency response of the IF bandpass filter (BPF) is determined by IMRR, the in-band flatness. The designed IF BPF adopts the Butterworth type with excellent in-band flatness. The desired Butterworth filter order can be calculated by (1).

(1) $$\begin{array}{c} N=int\left[\frac{log_{10}^{\left(2\;\times \;10^{\left(AL/10\right)}-1\right)}}{2log_{10}^{\left(f/f_{c}\right)}}\right]+1, \end{array}$$

where *N* is the order, and $f_{c}$ is the −3 dB bandwidth frequency, which in this design is equal to 2.046 MHz. *f* is the frequency difference between a specific frequency ($f_{sp}$) and the central frequency. AL is the out-of-band attenuation at $f_{sp}$. Considering the flicker noise and DC offset, the central frequency of this paper is 4.092 MHz [20,21]. For instance, the difference between the mirror frequency and the center frequency is 8.184 MHz. In other words, for IMRR, the frequency difference *f* is 8.184 MHz. As mentioned in Section 2, an IMRR of 25 dB is required. It means that at the mirror frequency, the out-of-band attenuation AL is 25 dB. So, according to (1), the needed filter order N is 3 to meet the IMRR requirement.

## 4. Receiver Design

### 4.1. Chip Architecture of the Receiver

**Figure 2:**
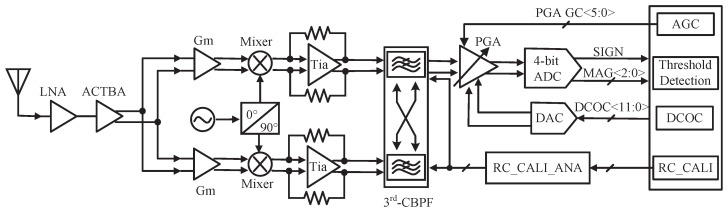
Receiver diagram.

The diagram of the receiver applicable to BDS-3/GPS, working in the L1 band at 1575.42 MHz, is shown in Figure 2, where all blocks are integrated into the chip. The receiver adopts a single conversion low-IF structure, rather than a zero IF structure, to reduce the impact of a higher flicker noise [4,7]. LNA is the first stage connected to the antenna, so it dominates the noise behavior of the entire receiver [5]. The input of LNA matches 50 Ω in the narrow band near the navigation frequency of 1575.42 MHz. The subsequent ACTBA converts the single-ended RF signal into differential signals to avoid the common-mode noise. Compared with the structure of a passive balun plus a differential amplifier, a certain gain is provided by ACTBA to reduce the gain pressure of other RF stages and save the area with comparable power. A passive switching mixer with a smaller area is used to down-convert the RF signals to IF signals using differential LO signals from the frequency synthesizer (FS). The maximum gain of a passive switching mixer is only 22ππ. Therefore, Gm, the passive switching mixer, and Tia form the GMT circuit to compensate for RF gain. The down-converted IF signal is then filtered by a third CBPF with a programmable bandwidth of 2 or 4 MHz, providing image rejection, and it is easy to integrate. An RC calibration is used in CBPF to avoid the influence of process variation on the center frequency and bandwidth. A two-stage PGA with a digital AGC loop is employed to ensure that the signal magnitude at the ADC input is constant, regardless of the received signal strength. Due to the image rejection of CBPF, only the in-phase path signal is amplified and quantized to reduce the area and power by half. The DC offset correction (DCOC) circuit is adopted at the last stage of PGA to avoid signal saturation. Finally, the IF signals are digitized by a four-bit ADC. DCOC, AGC, and RC calibration are all implemented digitally, effectively reducing the chip area. Also, they are automatically shut down after convergence to effectively reduce power.

### 4.2. RF Front-End

**Figure 3:**
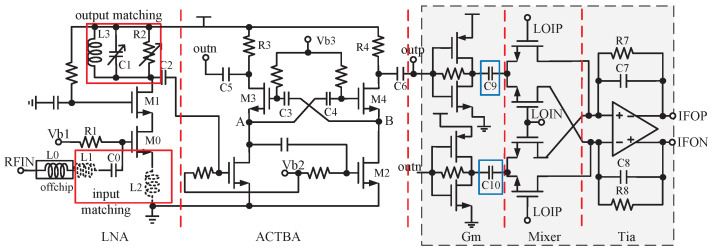
RF front-end circuit where $L_{1}$ and $L_{2}$ represented by dashed line are realized by bonding wires.

The RF front-end includes LNA, ACTBA, and GMT circuits, shown in Figure 3. Considering the cost, this design aims to avoid using an off-chip LNA when the interference is weak. Therefore, the on-chip LNA must have sufficiently low NF and high gain. For navigation frequency, if the source inductor of LNA is spiral, a large area of 220 μm × 220 μm is needed. An active inductor wastes voltage headroom, deteriorating the amplifier’s large signal output capability, and introducing noise. Therefore, inductors $L_{1}$ and $L_{2}$ are realized by bonding wires [5], which are represented by dotted lines in Figure 3. The simulated values of $L_{1}$ and $L_{2}$ are 2 nH and 0.6 nH, respectively. The length of the bonding wire corresponding to 2 nH seems to be long, but $L_{1}$ and the off-chip inductor $L_{0}$ are connected in series; this long length can be reduced by increasing $L_{0}$. The die position and the height of the bonding wires can be controlled on the package, thus controlling the length of the bonding wires in the range of ±10%. At the matching frequency, (2) holds.

(2) $$\begin{array}{c} \omega =2\pi f=\sqrt{\frac{1}{\left(L_{g}+L_{s}\right)C_{\mathrm{gs}0}}}, \end{array}$$

$L_{g}$ is the gate inductance, $L_{s}$ is the source inductance, and $C_{gs0}$ is the parasitic gate–source capacitance of $M_{0}$ in Figure 3. According to (2), f varies from −1.98% to 2.17%, which is acceptable. The cascode circuit composed of $M_{0}$ and $M_{1}$ can effectively reduce the LO leakage to the antenna. The output-matching network is composed of $R_{2}$, $C_{1}$, and $L_{3}$, where $R_{2}$ and $C_{1}$ are reconfigurable; the LNA has an improved in-band gain and higher out-of-band rejection than the traditional resistive load [8]. $R_{2}$ is used to adjust the Q of the resonant network at the output of the LNA, thereby adjusting the gain. That is, if the measured maximum gain of the entire system is low or high, the gain of the LNA can be manually adjusted through $R_{2}$ at the factory. We use an inductor instead of a current mirror, as the load consumes a very small voltage margin and achieves a higher resonant load, thus improving linearity and reducing power. $L_{3}$ is a spiral inductor with a quality factor of 11.9 and a value of 5 nH. However, the resonant frequency of this output-matching network changes with the variation of the corner and temperature. Therefore, the resonant frequency needs to be corrected by adjusting $C_{1}$ when leaving the factory. The simulation results show that the NF of the on-chip LNA is 1.3 dB with high gains of 23.7 dB and $IP_{1\mathrm{dB}}$ is −12.92 dBm.

The ACTBA [8] shown in Figure 3 follows the LNA through the capacitor $C_{2}$ with a value of 670 fF and converts the RF signals into differential signals, which are then down-converted to in-phase and quadrature low-IF differential signals by GMT. The cross-coupled structure composed of $M_{3}$, $M_{4}$, $C_{3}$, and $C_{4}$ can reduce the noise from $M_{2}$ [14,22], and further reduce the Miller effect from the input transconductor stage of the ACTBA [8]. Ignoring the channel length modulation and body effects, the differential signal with equal amplitude and phase difference of 180° can be obtained at output nodes outp and outn, as long as $g_{m3}R_{3}=g_{m4}R_{4}$ holds. In this design, $R_{3}$ and $R_{4}$ are equal to 1.144 kΩ. A certain phase error will be introduced. However, by reasonably adjusting the size of the devices in the ACTBA, the phase error between outp and outn can be controlled to less than $0.2^{\circ }$, which does not affect the navigation performance. The simulation results show that the gain of the ACTBA is 18.2 dB, and the cascaded NF with LNA is 1.3 dB.

The differential mixer is used to suppress common-mode interference. In Figure 3, the signals of ACTBA are transmitted to Gm through capacitors $C_{5}$ and $C_{6}$ with the same value of 2.216 pF; Gm, mixer, and Tia make up GMT. A passive switching mixer is selected due to its high linear characteristics and low noise performance [23]. A self-biased inverter structure and current reuse technology [7] are used in the Gm circuit to improve the gain while reducing power. The Tia is similar to that in [8]. The Gm and Tia can provide a certain gain to relieve the gain pressure of other RF stages. In the traditional blocking scheme, the blocking capacitors are located at the input of the CBPF. These capacitors are advanced to the output of Gm, described as capacitors $C_{9}$ and $C_{10}$, with the same value of 2.216 pF in Figure 3. The frequency is higher than that at the input of the CBPF, so the capacitors are smaller and fewer, conserving space. The simulation shows that for the RF front end, the cascade gain is 52.3 dB and the cascade NF is about 1.5 dB. There are magnitude and phase imbalances in the differential output of ACTBA. However, because of the structure of the mixer, the input mainly focuses on the difference between the positive and negative signals. Therefore, even if the ACTBA outputs have imbalances, the differential output of the IF will have good balance.

### 4.3. CBPF

The signal after GMT is centered at 4.092 MHz, and BDS-3/GPS signals are buried below the thermal noise. For BDS-3/GPS, the required bandwidth is 4.092/2.046 MHz, respectively. In order to realize a complex filter, a third-order real low-pass filter needs to be realized first, and then the IF shift is realized by quadrature coupling [24]. In our design, taking the third-order RLC low-pass filter as the prototype, a third-order Butterworth-type in-phase and quadrature (IQ) CBPF is realized by leapfrog. There are DC-blocking capacitors at the output of the Gm, so the RF common-mode (CM) voltage is not transmitted to the mixer. In addition, there is a common-mode feedback (CMFB) circuit in the Tia. Therefore, the CM voltage of the Tia is the DC operating point of the mixer. Moreover, the CMFB circuit of the later-stage CBPF is the same as that of Tia. So the CM voltages of these two sub-circuits are the same. There is no need for blocking capacitors between GMT and CBPF. Moreover, compared with the traditional method of adding DC-blocking capacitors to the CBPF input, this method uses fewer capacitors and a smaller capacitance value, thus conserving space.

RC calibration is employed to adjust the bandwidth and IF digitally [25] to avoid suffering from process changes. Under different simulation corners, the resistance and capacitance values vary by ±15% and ±10%, respectively. This will cause a deviation of −23.5%∼+26.5% in the RC time constant. Thus a deviation of −20.95%∼+30.72% in the center frequency will be observed without the RC calibration. Such a large deviation will cause useful signals to be greatly suppressed. To ensure that the bandwidth of CBPF can cover the useful signals without loss under the process, voltage, and temperature (PVT) variations, the center frequency deviation range should be controlled within ±2%, so the RC calibration accuracy should be within the range of −1.96%∼+2.04%. To reserve a certain margin, the RC calibration accuracy is set within ±1.8% in this design.

**Figure 4:**
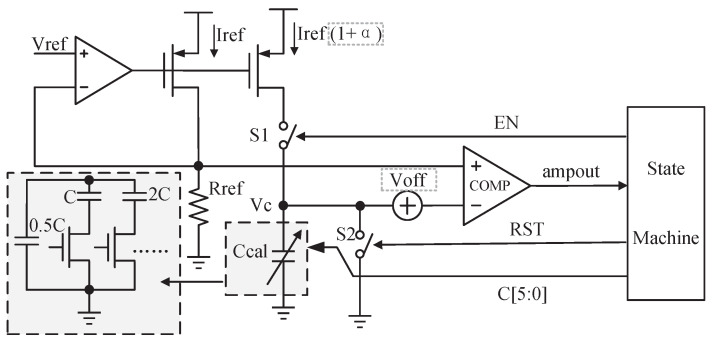
Diagram of the RC calibration.

The RC calibration diagram is shown in Figure 4. Firstly, the RST signal is enabled to discharge the calibration capacitor $C_{cal}$ until its voltage $V_{c}$ equals zero. Then RST is disenabled, and the signal EN is enabled and lasts $T_{C}$. The reference current $I_{ref}$ is converted from the reference voltage $V_{ref}$ and is equal to $V_{ref}/R_{ref}$. After a current mirror, this current is used to charge $C_{cal}$ when EN is enabled. $C_{cal}$ is a replica of the capacitor in CBPF. The charged voltage $V_{c}$ is compared with $V_{ref}$ to generate the signal C[5:0] controlling $C_{cal}$. At the calibration beginning, $C_{cal}$ is the smallest and then increases gradually. A process—such as the discharge, charge, and comparison—is required for each increase. In the beginning, $V_{c}$ exceeds $V_{ref}$ within $T_{C}$, making the signal ‘ampout’ low. As $C_{cal}$ increases, the $V_{c}$ obtained within TC becomes smaller until it is equal to $V_{ref}$. At this point, the ’ampout’ reverses to high and the calibration concludes, holding (3).

(3) $$\begin{array}{c} R_{ref}C_{cal}=T_{C}, \end{array}$$

With C[5:0] obtained at this point, the desired RC is achieved. There is a mismatch in the current mirror, which is represented by α in Figure 4. There is a DC offset voltage, $V_{off}$, of the comparator. Both factors will introduce errors to the RC time constant, and the error can be expressed as

(4) $$\begin{array}{c} {RC}_{\mathrm{error}}=\frac{{\alpha V}_{ref}-V_{\mathrm{off}}}{V_{ref}+V_{off}}T_{c}\approx \left(\alpha -\frac{V_{\mathrm{off}}}{V_{ref}}\right)T_{C}, \end{array}$$

**Figure 5:**
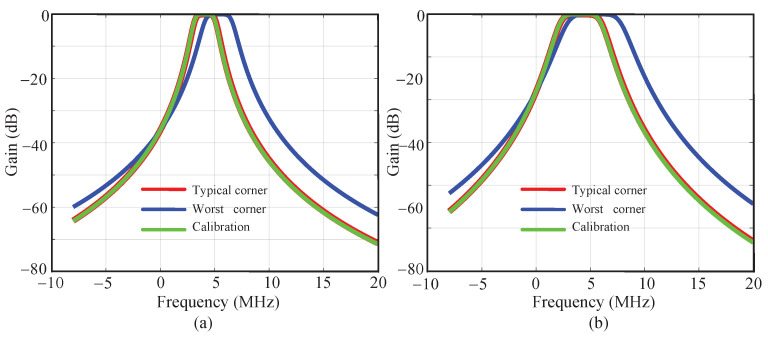
Comparison of the frequency response before and after the RC calibration for the bandwidth of (**a**) 2 MHz and (**b**) 4 MHz.

As mentioned above, the simulated deviation in the RC time constant is −23.5%∼+26.5%. In the design, the change of $C_{cal}$ is considered as ±30%. The capacitors in the $C_{cal}$ array are incremented in binary and the control signal is 6 bits. So the error caused by the unit capacitor of $C_{cal}$ is (0.3−(−0.3))0.3−(−0.3))(2(26−1)=0.95%. By adding a half-value fixed capacitor as shown in Figure 4, an accuracy of 0.475% is realized in the calibration capacitor array. And the 3σ correction errors of α and $V_{off}$ are 0.5% and 0.8%, respectively. The three items constitute the RC calibration accuracy of ±1.8%. To verify the performance of the RC calibration, the receiver works at the worst corner where the resistors and capacitors are at the ff corner and −40 °C, showing a maximum shift in the center frequency and bandwidth. Then the RC calibration process is enabled. The calibrated frequency response is compared with that under the typical corner, and the simulation results are shown in Figure 5. It proves that the RC algorithm realizes a perfect calibration of the center frequency and bandwidth.

The CBPF has two operating modes: the narrowband mode for GPS and the broadband mode for BDS-3/GPS. The simulation results show that the bandwidths are, respectively, 2.046 MHz and 4.092 MHz, and there is no ripple in the band. IMRRs are greater than 50 dB and 45 dB, respectively. The simulated $IP_{1\mathrm{dB}}$, referred to as the antenna input, is −43.54 dBm with an NF of 32.5 dB, meeting the system requirements.

### 4.4. AGC and DCOC

**Figure 6:**
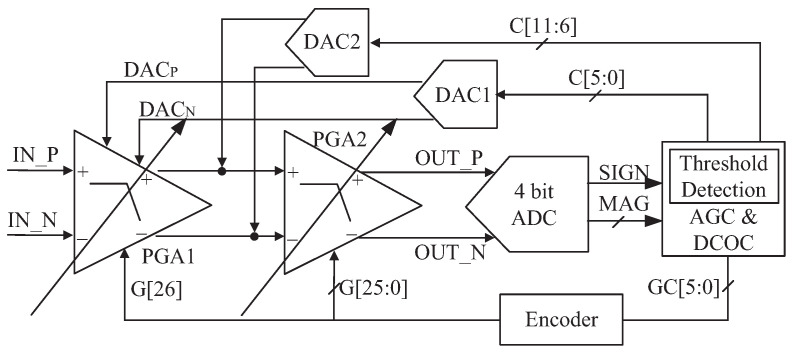
Simplified AGC and DCOC circuit diagram.

**Figure 7:**
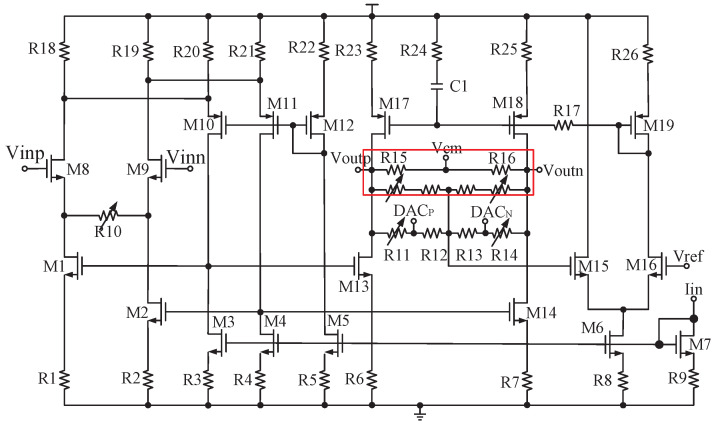
PGA circuit where the resistors in the red box are owned only by PGA2.

Although the out-of-band interference is attenuated, the spectrum after CBPF is still determined by the in-band thermal noise. Therefore, the main purpose of AGC is to minimize the error between the IF output power and ADC full-scale power [20]. DCOC is necessary to avoid reception failure due to signal saturation [7]. The receiver only supports a single frequency of 1575.42 MHz for BD3 and GPS, and only the in-phase path output of CBPF is amplified and processed, so the area and power are reduced a lot. The simplified AGC and DCOC circuit diagram is shown in Figure 6, where PGA is the key to a sufficiently wide gain range [5]. SIGN and MAG are, respectively, the symbol and amplitude of the four-bit ADC output. The PGA circuit composed of a two-stage differential amplifier with a common-mode feedback circuit is shown in Figure 7, whose gain can be changed by $R_{10}$, $R_{11}$, and $R_{14}$. PGA1 differs from PGA2 in that the resistors in the red box in Figure 7 are only owned by PGA2. GC[5:0] is the gain control code, encoded as G[26:0]. The most significant bit (MSB) G[26] controls $R_{10}$, $R_{11}$, and $R_{14}$ in PGA1 for coarse tuning, G[25] controls $R_{11}$ and $R_{14}$ in PGA2 for coarse tuning, and G[24:0] controls $R_{10}$ in PGA2 for fine-tuning. C[11:0] is the offset control code. The 6 MSBs C[11:6] are connected to the output of PGA1 through DAC2 for coarse tuning and the 6 least significant bits (LSBs) C[5:0] are connected to the internal nodes of the PGA1 (i.e., $DAC_{P}$ and $DAC_{P}$ in Figure 7) through DAC1 for fine-tuning. By adjusting these four terminals, the DC offset voltages of $V_{outp}$ and $V_{outn}$ can be changed to achieve DCOC. The two control codes are generated by detecting MAG and SIGN signals, respectively. In Figure 7, $R_{18}$∼$R_{22}$ have the same value, and so do $R_{3}$∼$R_{5}$. $M_{3}$∼$M_{5}$ have the same W/L and so do $M_{10}$∼$M_{12}$. On this basis, the bias current of $M_{8}$ and $M_{9}$ is determined by the bias current of $M_{5}$. $M_{10}$ is connected to $M_{1}$, forming a negative feedback loop to stabilize the first stage output. Compared with the traditional folded cascode amplifier, this structure saves two bias circuits (the bias currents of the input transistors and the bias currents of $M_{1}$ and $M_{2}$), thus conserving space and power.

Since the signal received by the navigation system is a fixed thermal noise power, the gain can be fixed once AGC is completed. Therefore, there is no need for a table to store gains, saving chip memory. In cycle 512CLK, ADC outputs MAG at the falling edge of the clock, while the AGC algorithm samples it at the rising edge and counts the results to obtain the actual threshold value $M_{AGC}$. Two power thresholds are configured by the serial peripheral interface (SPI), which are, respectively, the high threshold powth_high and the low threshold powth_low in the power range. When navigation signals are received, if $M_{AGC}$ exceeds the power range, AGC will be enabled. $M_{AGC}$ is then compared with the average value of the two powers powth_mid ((powth_high+powth_low)(powth_high+powth_low)22). If $M_{AGC}$ is larger than powth_mid, the PAG gain decreases and then $M_{AGC}$ is recalculated and recompared with powth_mid. Until $M_{AGC}$ is smaller than powth_mid, the gain is stable, and vice versa. After that, as long as $M_{AGC}$ is within the power range [powth_low, powth_high], the gain will remain unchanged. This is mainly to reduce the influence of the detection accuracy and signal randomness on the $M_{AGC}$ calculation. The duty cycles of the MSB MAG signals are 33% and 18.85% for two- and four-bit ADCs, respectively, to provide a negligible SNR loss that is less than 0.5 dB [5,8], which is used to determine powth_high and powth_low.

**Figure 8:**
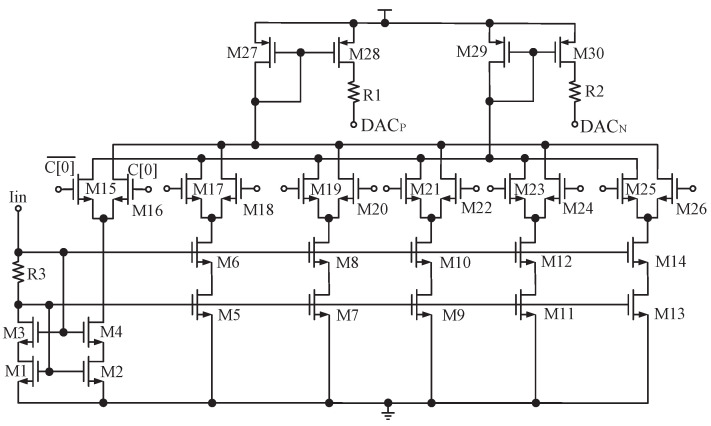
DAC circuit.

**Figure 9:**
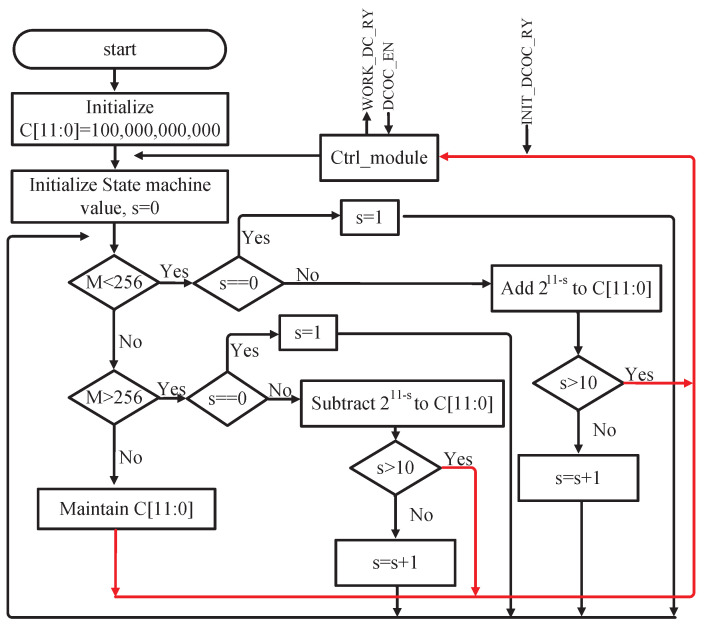
Flow chart of the dichotomy search method for DCOC.

DCOC is implemented based on DACs, as shown in Figure 6. There is no operational amplifier in DAC and the currents of the current source array directly flow through resistors to improve speed and reduce power, as shown in Figure 8. DAC steers currents from PGA or releases currents to PGA by evaluating the signal SIGN to eliminate the DC offset of its balanced branches [14]. Similar to the AGC detection principle, the sum of SIGN is calculated within 512CLK, and recorded as $M_{DCOC}$. Ideally, the DC offset is 0, and $M_{DCOC}$ should be 256, with a duty cycle of 50%. When the receiver is powered on or the gain of the PGA is changed, DCOC is performed. The code C[11:0] starts from 100,000,000,000 and the dichotomy scan is used to make $M_{DCOC}$ approach 256. The dichotomy process for DCOC is shown in Figure 9, where s represents the bit number of C[11:0]; that is, C[11:0] from the most to the least significant bit corresponds to s equals 0, 1, 2,..., 11. For example, D[11] corresponds to s equals 0, and D[10] corresponds to s equals 1. When $M_{DCOC}$ equals 256 or s is beyond 10, the DCOC ends, as shown by the red line in Figure 9. In general, the probability of $M_{DCOC}$ being exactly 256 is very low, so C[11:0] corresponding to s beyond 10 is the code that makes $M_{DCOC}$ closest to 256.

The Monte Carlo simulation is carried out at the PGA1 outputs to verify the performance of the DCOC circuit. The mean output DC offset of 6.9 mV and the 3σ variation of −340 to 350 mV are observed without DCOC. This means that PGA1 amplifies the offset voltage of the previous circuits to more than 300 mV, which makes DAC need a wide output range. PGA2 amplifies the residual after DCOC to ADC; this residual amplified to ADC needs to be smaller than the accuracy of ADC, so a high-accuracy DAC is needed. As a result, two six-bit DACs are adopted, one for coarse tuning and the other for fine-tuning, as mentioned above. Since the DAC is differential and the sum of the output currents of the DAC remains constant, the currents from DAC do not affect the common-mode voltage of the PGA outputs.

**Figure 10:**
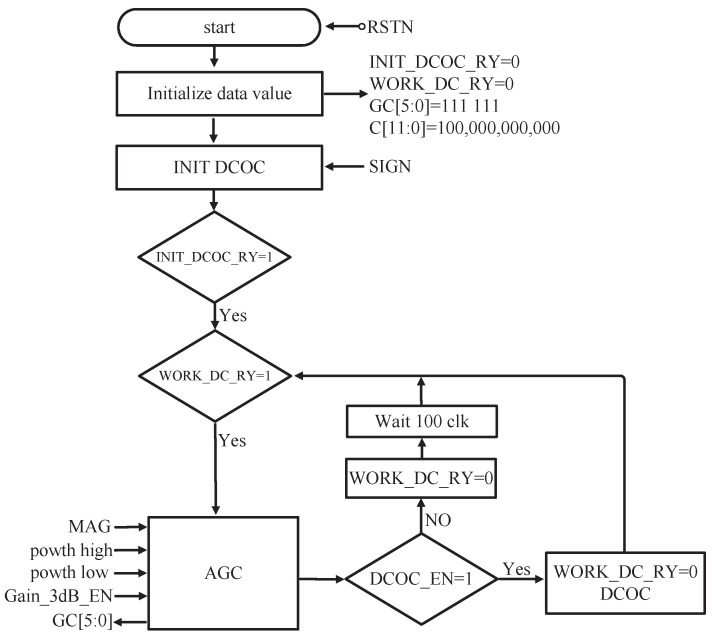
Mixed flow chart of AGC and DCOC.

**Figure 11:**
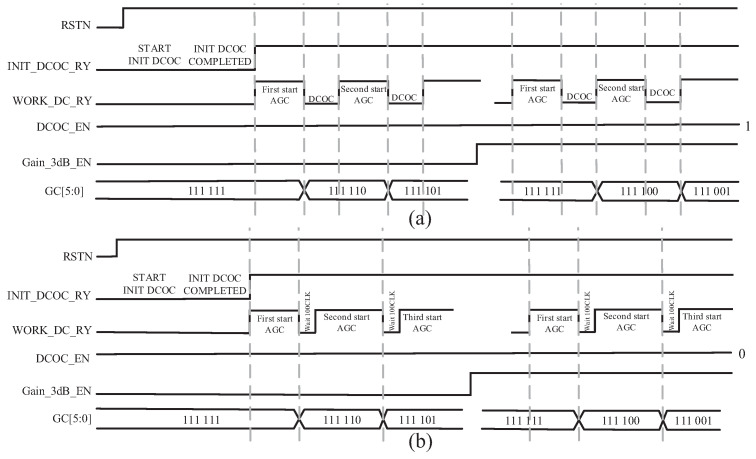
Timing diagram when (**a**) enabling AGC and DCOC, (**b**) enabling AGC and disabling DCOC.

When DCOC_EN is 1, if the gain control code GC[5:0] is changed, the signal WORK_DC_RY is set to 0 and DCOC is executed. When the DCOC is complete, we set WORK_DC_RY to 1 and start the next AGC. If DCOC_EN is 0, DCOC is not executed. So the signal WORK_DC_RY is first set to 0 and then set to 1 after 100CLK. Then we start the next AGC. The mixed flow chart of AGC and DCOC is shown in Figure 10. When powering up, we initialize the DCOC first; that is, the signal INIT_DCOC_RY is set to 0, and when the DCOC initialization is complete, the signal INIT_DCOC_RY is set to 1. At the same time, because the DCOC is complete, the signal WORK_DC_RY is set to 1. The signal Gain_3dB_EN determines whether the gain step is 1 dB or 3 dB. The timing diagram is shown in Figure 11.

A four-bit flash ADC [7] with a resistor ladder is adopted. The PGA’s differential outputs are shorted through resistors to obtain the common-mode voltage (i.e., $V_{cm}$ in Figure 7). To reduce the ADC’s quantization error, this common-mode voltage is used as the input of the resistor ladder. The simulation shows that the PGA can achieve a programmable gain range of 63.1 dB, and the output voltage range of DAC is ±398 mV, covering the DC offset range observed at outputs of PGA1 without DCOC; moreover, the compensation voltage accuracy is less than 0.2 mV.

## 5. Measurement Results

**Figure 12:**
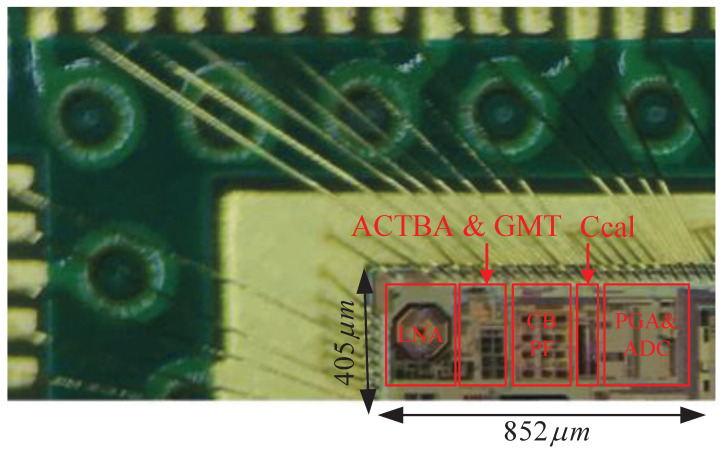
Chip-on-board bonding physical map with an active area of 852 μm × 405 μm.

This receiver is fabricated in a standard 55 nm CMOS technology; to shorten the research time and test more pins to facilitate the test, the chip is first directly bonded onto an evaluation PCB, and then housed into a quad flat no-lead (QFN) package. The chip-on-board physical layout, spanning an active area of 852 μm × 405 μm, is shown in Figure 12. Low-cost copper wires are used for bonding. In this design, the effect of package parasitism on the chip performance has been considered, so there is no significant difference in the performances of the two packages.

**Figure 13:**
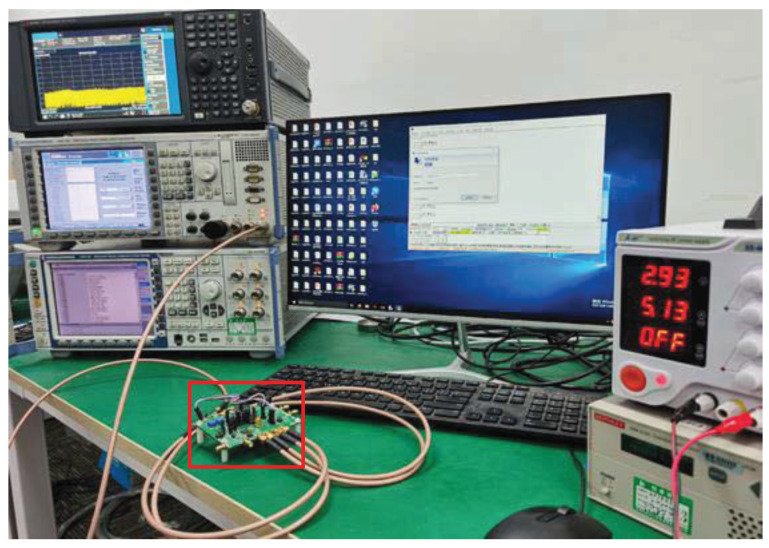
Hardware platform with the test board.

**Figure 14:**
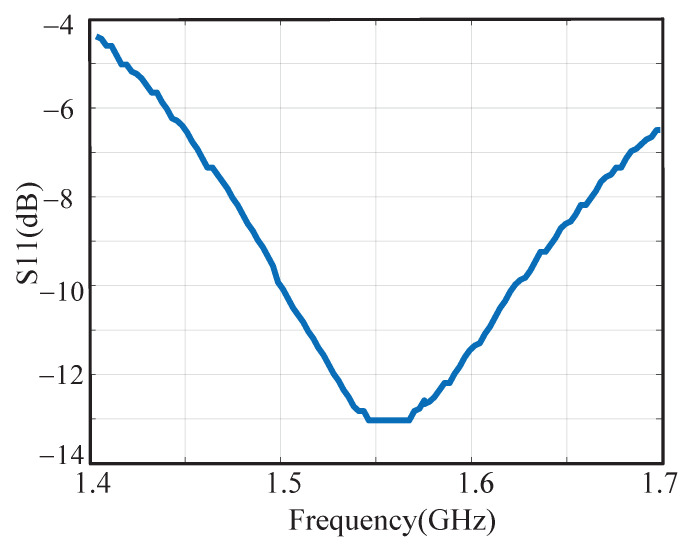
Measured input matching.

The hardware platform is shown in Figure 13, where the test board is in the red rectangle. The used equipment is presented in Table 2. The current is 4.8 mA from a 1.2-V supply, and the current consumption of sub-circuits is presented in Table 3. There are pre-amplifiers, a comparator, latches, and coding circuits in ADC, and the currents are 0.75, 0.055, 0.074, and 0.021 mA, respectively. The receiver realizes a good input matching with $S_{11}$ around −13 dB at 1575.42 MHz, as shown in Figure 14.

**Figure 15:**
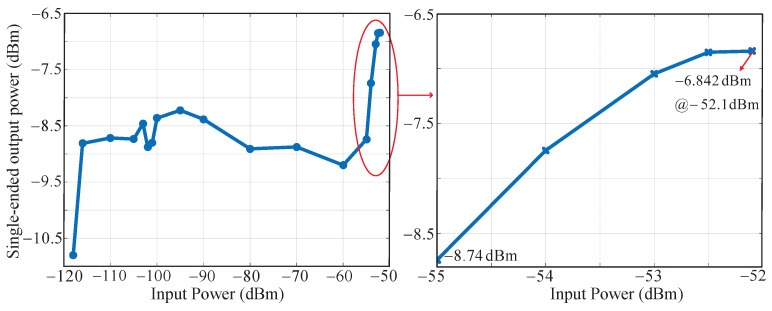
Single-ended output power (dBm) versus input power (dBm).

As shown in Figure 15, when the input power is from −116 dBm to −55 dBm, the IF single-ended output power is about −8.5 dBm, indicating that the Gr is 61 dB. When the input power is −118 dBm, the receiver achieves a maximum gain of 113.2 dB. In addition, when the input powers are −55 and −52.1 dBm, the single-end output powers are −8.74 and −6.842 dBm, respectively, and the cascaded gains of 52.26 and 51.258 dB are obtained. So the measured $IP_{1\mathrm{dB}}$ is about −52.1 dBm.

**Figure 16:**
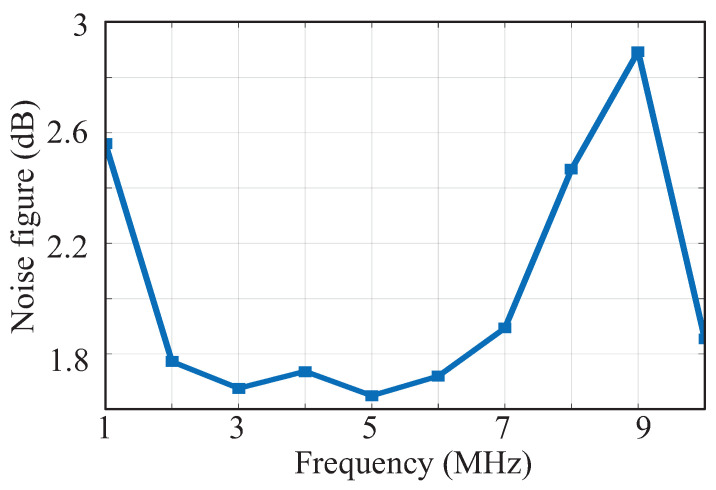
Measured noise figure.

The measured noise figure is described in Figure 16, showing that NF is 1.74 dB at 4 MHz, which is much better than the requirement presented in [10]. When the interference is not weak, as mentioned above, a SAW filter, such as Murata’s SAFFB1G57KE0F0A, is needed after the antenna. It uses physical methods to realize the filtering function and does not consume the current. In order to prevent the SAW filter from worsening the NF, an external monolithic LNA, Silicon Wave’s SW7121D, will be added in front of the SAW, whose NF is 0.9 dB with a gain of 16 dB and a current of 1.2 mA. Therefore, the NF of the monolithic LNA and SAW almost does not worsen the NF of the whole system. In addition, the whole NF will not be higher than 0.95 dB.

**Figure 17:**
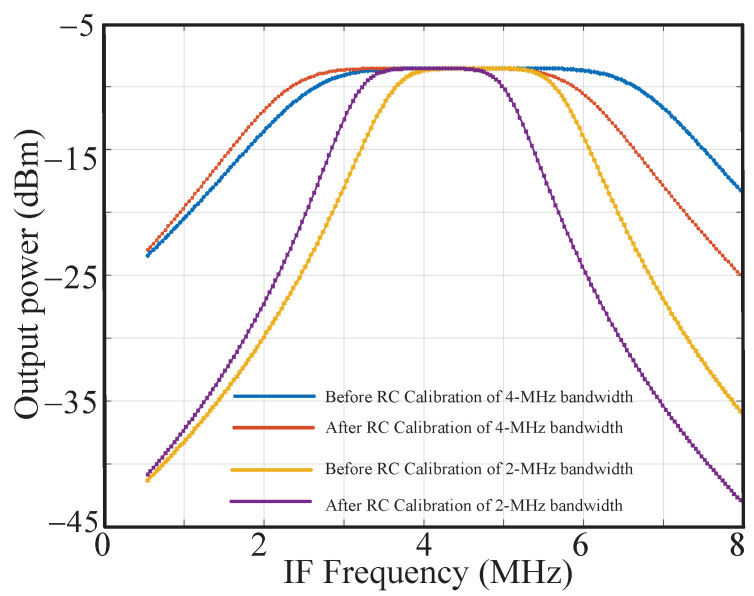
Measured performance of RC filter calibration.

**Figure 18:**
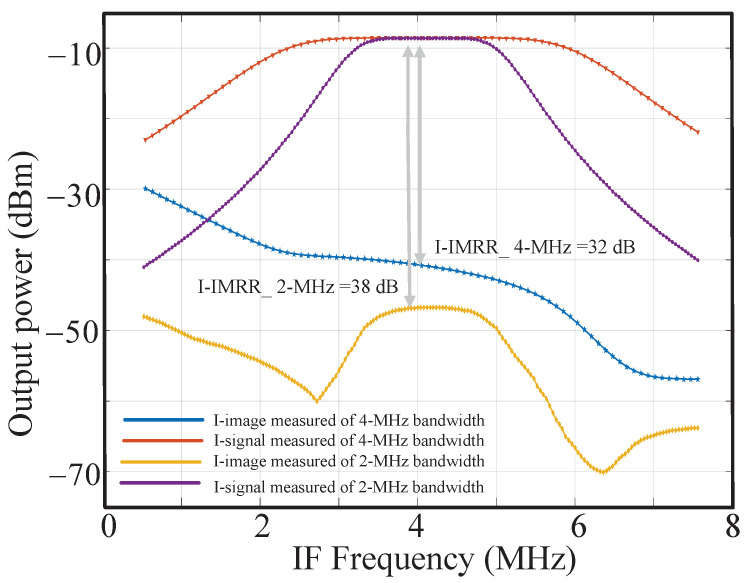
Measured signal and image response.

The performance of the RC calibration is measured at the PGA’s output, as shown in Figure 17. It can be seen that the center frequency and bandwidth can be well calibrated in the case of a 2 MHz or 4 MHz bandwidth. Figure 18 shows the signal and image response in the I channel of the 2 MHz and 4 MHz bandwidths. The IMRRs are 38 dB and 32 dB, respectively. The navigation signal source GNS8110 is used to generate the navigation signal as the input of this chip, and its performance is tested under normal reception. High tracking sensitivity makes code and carrier tracking loops more robust and positioning more reliable in weak signal environments, such as cities and canyons [26]. The tested acquisition sensitivity, tracking sensitivity, position accuracy, velocity accuracy, position update rate, and reacquisition time are −148 dBm, −159 dBm, 0.56 m, 0.025 m/s, 10 Hz, and 1.94 s, respectively.

**Figure 19:**
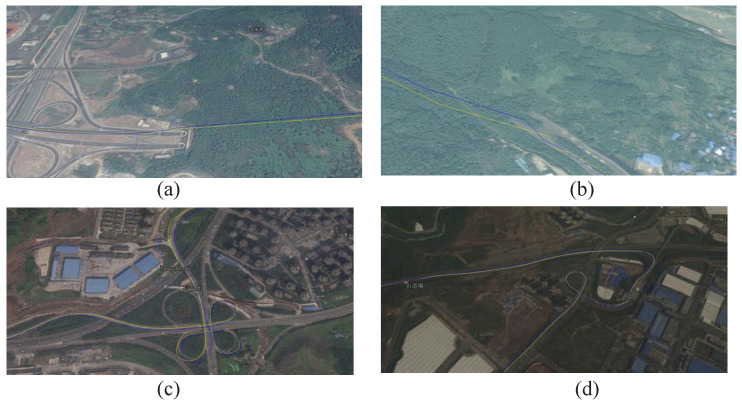
Measured positioning results of (**a**) broad road, (**b**) tunnel road, (**c**) overpass road, (**d**) curve road.

After the in-laboratory test, outdoor tests, including the broad road, tunnel road, overpass road, and curve road, were carried out. The measured navigation tracks are compared with the navigation module at the leading level, as shown in Figure 19, where the yellow and blue lines are the tracks of the navigation module and this designed chip, respectively. It should be noted that when the navigation signal is weak, there will be a slight position offset. When the navigation signal is strong with less occlusion, the position is accurate. The trend is consistent with that of the reference navigation module. These results provide convincing evidence that the proposed RF receiver can be well-suited for navigation applications.

Table 4 summarizes the performance of the proposed receiver that is suitable for both BDS-3 and GPS in the L1 band in comparison to other reported research. In this work, in the on-chip LNA, the source inductor and gate inductor with high Q are realized by a copper bonding wire, improving the in-band noise performance and conserving space. LNA adopts a cascode amplifier structure with the LC resonant network as the load and with reused current, improving the gain and reducing the power consumption. The ACTBA, instead of the traditional passive balun, is adopted to convert the single-ended signal into the differential signal; this not only improves the RF front-end gain but also reduces the gain-realization difficulty of the later-stage circuit, which is conducive to achieving high gain and conserving space throughout the whole system. ACTBA also incorporates current reuse to reduce power consumption. A GMT circuit is modified, which can save the IF DC-blocking capacitors to reduce the insertion loss and area. The specific approach is to advance the DC-blocking capacitor originally located at the input of the CBPF to the output of the Gm so as to use fewer capacitors and a smaller capacitance value, thus conserving space. Image suppression is realized by BPF, and the later circuits only amplify and convert the in-phase path signals, saving nearly half of the IF layout area. Most of the RF front-end gain is realized by the IF PGA, which incorporates an open-loop circuit and has high-gain and low-power characteristics. After convergence, AGC and DCOC shut down automatically, effectively reducing power.

A fractional-N phase-locked loop (PLL) as the FS [27] is integrated into the same chip, which is not discussed in this paper. For a fair comparison, the area and current consumption of the FS are included in Table 4, and the area and current of GPS only or BDS only are summarized. This work succeeds in minimizing area, current, and NF. The maximum gain is better, except for [28]. The linearity of this work does not have much advantage over other references, but it is sufficient to meet the system requirements mentioned in Section 3. There is no other interference except for the natural thermal noise or other transmitter leakage noise near the image frequency. This noise power is quite low, so the requirement for IMRR is not strict. Although the IMRR performance of this paper is not superior to other references, it still ensures full compliance with the requirements of navigation specifications [10]. The FOM in Table 4 shows that this work is superior when considering the gain, NF, power, and area. In summary, a compromise is made among power, area, image rejection, and linearity in this work.

**Table 4:** Performance summary and comparison table.

Parameter	This Work	Ref. [28]’20	Ref. [7]’22	Ref. [29]’23	Ref. [30]’20	Ref. [31]’18
Tech. (nm CMOS)	**55**	110	65	28	180	180
Integration Level	**Fully**	Fully	Fully	Fully	Fully	Fully
Frequency Bands	**L1/E1**	L1/E1/L5/E5	L1/L2/L5/S	L1/L2/B1/E1	L1/B1/E1	L1/B1/E1
Navigation Systems	**GPS/BDS3/ Galileo**	GPS/BDS2/ Galileo/ GLONASS/ QZSS/IRNS	NavIC/GPS/ Galileo/BDS2	GPS/BD2/Galileo	GPS/BD2/Galileo	GPS/BD2/Galileo
NF (dB)	**1.74**	2.3	3.8∼4.4	3.1	1.79	1.8
Bandwidth (MHz)	**2/4**	2∼52	2/4/17/20/24	2/4	2/4	2/4
Max gain (dB)	**113.2**	131 ^1^	101.7	103.8	108	107.2
Gr (dB)	**61**	-	53.53	94.6	56	78
IMRR (dB)	**32**	-	28 (I channel) 42 (Q channel)	60.3	51.46 (Max) 32.98 (Min)	39.1
$IP_{1\mathrm{dB}}$ (dBm)	**−52.1**	-	−52 (L5 Band) −56 (S Band)	-	-	−29
IIP3 (dBm)	**−43.39**	-	−38 *	−9.9 (2M-BW) −4.5 (4M-BW)	−18.09	−19.325
Area (mm^2^)	**0.345**	-	0.83 ^2^	1.315 ^4^	0.79 ^6^	0.61 ^8^
Area (RX+FS) (mm^2^)	**0.73**	2.25	1.25 ^3^	-	1.64 ^6^	1.36 ^8^
Current (mA)	**4.8**	9.62 ^1^	24.08	32.8 ^5^	-	-
Current (RX+FS) (mA)	**8.7**	14.12 ^1^	38.09	-	16 ^7^	16
Supply Voltage (V)	**1.2**	1.5	1.2	1.1	1.8	1.8
$FOM1{\;}^{\#1}$	**108.48**	-	84.1	83.94	-	-
$FOM2{\;}^{\#2}$	**105.9**	-	82.11	-	92.64	92.95

- No Information Available. * Calculated according to the simulation results of LNA, mixer, and CBPF. ^1^ Pre simulation. ^2^ The total area of the circuits in the white box is 1.25 mm^2^ without a padring. The layouts of VGA, CBPF, LNA+MIXER, and ADC are assumed to be compact, so the area excluding PLL, SPI, and BGR is estimated to be 0.83 mm^2^ (the area is larger than 0.83 mm^2^). ^3^ Without a padring. ^4^ It excludes CBPF2 and CBPF3, so it is only for GNSS and it excludes pads. ^5^ It excludes CBPF2 and CBPF3, so it is only for the GNSS, and the divider dynamic power consumption of 0.545 mW/GHz is not included. ^6^ It only includes one CBPF, one PGA, and one ADC for a single channel with ESD and pads. ^7^ It consists of two CBPFs, two PGAs, and two ADCs for dual channels. ^8^ With ESD and pads. ^#1^ $FOM_{1}$ = 10log (gain/(NF × power (mW) × area (mm^2^))), modified according to [32]. ^#2^ $FOM_{2}$ = 10log (gain/(NF × power (RX+FS) (mW) × area (mm^2^))), modified according to [32].

## 6. Conclusions

The receiver with the digital RC calibration, AGC, and DCOC presented in this work is used for processing navigation signals from BDS-3 and GPS. The receiver is fabricated in 55-nm CMOS technology, occupying an active die area of 0.345 $mm^{2}$ with RX only. The current consumes 4.8 mA with a supply of 1.2 V. The noise figure performance is excellent (only 1.74 dB). The current consumption and area are competitive. Compared with the advanced navigation modules in the market, the positioning performance of the receiver is verified, and the comparison result provides convincing evidence that the proposed receiver can be well-suited for navigation applications.

## Figures and Tables

**Table 1:** Requirement breakdown of each sub-circuit.

Sub-Circuit	Input	LNA	ACTBA	GMT	BPF	PGA
Max Gain (dB)		23	18	10	0	63
Min Gain (dB)		23	18	10	0	0
NF (dB)		2.3	8	13.5	35	35
IBP (dBm)	−75	−52	−34	−24	−24	−24
OBIP (dBm)	−56	−33	−15	−25	−100	−100
$IP_{1\mathrm{dB}}$ (dBm)		−23	−18	−10	0	0
IIP3 (dBm)		−13	−8	0	10	10

**Table 2:** List of the used equipment.

Name	Type	Name	Type
Galvanometer	N6705C	Vector signal source	SMBV100B
Navigation signal source	GNS8110	Frequency spectrometer	N9020B
Vector network analyzer	ZND	Frequency meter	53220A
Oscilloscope	DSOX6004A		

**Table 3:** Measured current consumption of sub-circuits with 1.2-V supply.

Sub-Circuit	LNA	ACTBA	GMT	CBPF	PGA	ADC
Current (mA)	1.2	0.5	0.6	0.9	0.7	0.9

## Data Availability

Not applicable.

## Abbreviations

The following abbreviations are used in this manuscript:

MDPI	Multidisciplinary Digital Publishing Institute
PVT	Process, voltage, and temperature
BDS-3	BeiDou navigation satellite system-3
GPS	global positioning system
PNT	positioning, navigation, and timing
IoT	Internet of Things
GLONASS	global navigation satellite system
IMRR	image rejection ratio
LNA	low-noise amplifier
ACTBA	active balun amplifier
DC	direct current
CBPF	complex bandpass filter
Gm	transconductance amplifier
RHCP	right-hand circular polarization
LO	local oscillator
IF	intermediate frequency
NF	noise figure
Tia	Transimpedance amplifier
ADC	analog–digital conversion
Gr	gain control range
$IP_{1\mathrm{dB}}$	input 1 dB compression point
IIP3	input third-order intercept point
IBP	in-band power
OBIP	out-of-band interference power
PGA	programmable gain amplifier
BPF	bandpass filter
FS	frequency synthesizer
DCOC	DC offset correction
IQ	in-phase and quadrature
CM	common-mode
CMFB	common-mode feedback
MSB	most significant bit
LSB	least significant bit
SPI	serial peripheral interface
QFN	quad flat no-lead
PLL	phase-locked loop
